# The medication self-management work system of patients and informal carers from a human factors & ergonomics perspective: A scoping review protocol

**DOI:** 10.12688/hrbopenres.13674.1

**Published:** 2023-01-10

**Authors:** Eduard Negoescu, Romaric Marcilly, Samuel Cromie, Aaron Koay, Tamasine Grimes

**Affiliations:** 1 School of Pharmacy and Pharmaceutical Sciences, Panoz Institute, Trinity College Dublin, The University of Dublin, Dublin, D02 PN40, Ireland; 2 Univ. Lille, CHU Lille, ULR 2694 - METRICS : Évaluation des technologies de santé et des pratiques médicales, Lille, F-59000, France; 3 INSERM, CIC-IT 1403, Lille, F-59000, France; 4 Centre for Innovative Human Systems (CIHS), School of Psychology, Aras an Phiarsaigh, Trinity College Dublin, The University of Dublin, Dublin, Ireland; 5 Institute for Global Health, University College London, London, WC1N 1EH, UK

**Keywords:** Human factors and ergonomics, household, patient, drug therapy, medication error, systems-based analysis, patient and public involvement, PPI

## Abstract

**Background: **Healthcare is increasingly delivered closer to the patients’ homes, which increases the level of responsibility that patients and informal carers take for managing their medication-taking, although this is associated with hazards. Medication self-management has been conceptualised as work taking place in non-formal settings ( *e.g.*, households), which are complex systems. Human factors and ergonomics (HFE) models provide a framework for studying such systems. The Systems Engineering Initiative for Patient Safety (SEIPS) is one framework that considers work system elements and how they interact with each other to shape processes that lead to outcomes ( *e.g.*, safety). Given the increasing amount of diverse research on patient and carer work and on system-shaping factors, the objectives of this review are to: (i) identify available evidence in a structured and systems-oriented way, (ii) explore approaches that have been applied and (iii) highlight research gaps.

**Methods:** An evidence-informed patient, public and carer involvement (PPCI) approach will be implemented at all post-protocol stages to ensure the relevance, uptake and translation of the scoping review. The review will systematically search MEDLINE, Embase, PsycInfo, CINAHL and Web of Science to identify relevant qualitative studies. The methodological approach will be guided by Johanna Briggs Institute methodology and will be reported according to the PRISMA-ScR standards. Data charting and qualitative content analysis directed by SEIPS will explore how the work system and its constituting elements have been described in the literature and identify specific gaps and opportunities for future research. Borrowing from realist approaches, included studies will be assessed in terms of richness and relevance to our review question.

**Discussion:** Strengths of this scoping review include PPCI and a converging focus on medication safety, medication self-management and HFE. Ultimately, this approach will advance our understanding of this complex system and guide opportunities to broaden and strengthen the evidence base.

## Introduction

There is a global drive to deliver healthcare as close to home as appropriate ^1, 2^, and to reduce exposure to unnecessary hospital admission. In this context, patients and their carers are taking on increasing levels of responsibility and burden of medication management ^3, 4^. Both positive and negative outcomes have been associated with lay medication practices in the home. For example, patients may sometimes successfully and conveniently integrate medication work into the structure of their daily lives ^3^. However, many hazards have been identified, including the sharing of prescribed medication ^5, 6^, non-adherence ^7^, hoarding ^8^, unsafe storage ^9^, inappropriate disposal ^10^, inappropriate dosage ^11^ and errors made by patients and their carers ^12, 13^.

This medication management by patients or their carers has been conceptualised as work, that is, effort expended to complete tasks associated with all aspects of medication use to achieve the desired beneficial effects and reduce the likelihood of undesirable effects ^3, 4, 14^. Such medication work is self-managed in non-formal settings ( *e.g.*, households), which are complex systems that can be explored using human factors and ergonomics (HFE) approaches. HFE seeks to understand how people and other elements within a system interact ^15^, and to apply systems-thinking models to “optimise human well-being and overall system performance” ^16^. The Systems Engineering Initiative for Patient Safety (SEIPS) is one such model ^17^. SEIPS considers how work system elements (people, tools, tasks, technologies, environments and their organisation) and the interaction between these elements shape work processes (at social, physical or cognitive levels), which in turn produce work outcomes that may strengthen or weaken safety. For instance, SEIPS has been used to explore household medication safety ^14, 18^. Further, a recent scoping review exploring contributory factors related to patient work identified a substantial number of publications on the topic of medication management ^4^, indicating that a dedicated review adopting a HFE perspective on the topic is needed.

Therefore, given the increasing body of work that patients and informal carers perform to self-manage medication in non-formal settings, there is a need to gather the evidence about this work in a structured, systems-oriented way, in order to identify research gaps and opportunities to advance research, policy and practice.

### The present need for research

Preliminary searches suggested the current body of literature is heterogenous, which reflects a need to unify and structure this knowledge to take a considerate HFE approach to study lay medication management. Moreover, the increasing prevalence and global burden of polypharmacy ^19^ add complexity to the work carried out by patients and carers in managing their medication. Finally, the need to research lay medication management is emphasised by the fact that pre-existing health inequalities can widen even more during public health emergencies such as the COVID-19 pandemic ^20^, as evidenced by outcomes such as medication-related harm.

## Objectives

This scoping review aims to describe and categorise the available evidence about non-formal medication self-management work systems. The scoping review aims to (i) use a HFE perspective to identify and structure studies about medication self-management as practiced by patients and informal carers in non-formal settings, (ii) to explore the data analytical approach applied in these studies and (iii) to identify gaps in the available body of evidence and opportunities for future research.

## Methods

### Patient, public and carer involvement

Patient, public and carer involvement (PPCI) in research is important to optimise the relevance and meaning of the research to those affected by the topic and to increase the uptake and translation of research into sustainable practice ^21, 22^. PPCI supports stakeholders, such as end users and professional or non-professional providers, to have parity with researchers. This helps stakeholders, patients and carers maintain a central status in the three domains of quality in healthcare, namely clinical effectiveness, patient safety and patient experience ^23, 24^.

This scoping review will incorporate and report on PPCI in line with the Authors and Consumers Together Impacting on eVidencE (ACTIVE) framework ^22^. Contributors will be invited to participate on a continuous basis at the following stages: selection of studies, data collection, qualitative analysis, interpretation of findings, writing and publishing, knowledge translation and impact, including outreach activities and the co-design of lay summary materials. A suitable level of engagement (leading, controlling, influencing, contributing or receiving) will be jointly decided with PPCI contributors identified on each occasion, having regard to their preferences and the resources available at each stage. Training will be provided to support the learning needs required to meet the tasks.

### Protocol development

The methodological approach is informed by scoping review guidelines published by the Joanna Briggs Institute (JBI) ^25^ and by recommendations on implementing PPCI according to the ACTIVE framework ^22^. Further, the reporting of this protocol observes the items on the Preferred Reporting Items for Systematic Reviews and Meta Analyses extension for Scoping Reviews (PRISMA-ScR) checklist that are relevant to a protocol ^26^. Additionally, a PRISMA extension for protocols (PRISMA-P) checklist ^27^ has been completed (access is detailed in the Data Availability section ^28^).

### Eligibility criteria

The eligibility criteria were developed around a modified version of the PCC tool (participants, concept, context) ^25^, presented below. Relevant peer-reviewed primary studies will be included while secondary studies will not, although references cited therein will be screened. This approach will help prevent inadvertent double counting of data originating from primary studies ^25^. There is no restriction on the date of study publication. We will attempt to translate studies published in languages other than English.

***Participants.*** Studies with a primary focus on lay participants such as patients and their non-professional carers ( *e.g.*, family members or laypeople who may or may not have received training) will be included. Studies focusing on healthcare practitioners, formally employed caregivers or professional carers will be excluded.

***Concept.*** Studies addressing any aspect of medication self-management are eligible for inclusion. For the purpose of this review, we consider medication self-management as any task undertaken by the patient, or their carer, for the purpose of using their medication, at any stage of the medication management process. Medication will be defined as an authorised allopathic product that may be prescribed or non-prescribed (available over-the-counter).

***Context.*** Studies exclusively addressing household, domestic or other non-formal healthcare settings will be included. Studies that have an element of professional or semi-professional service provision will be excluded.

***Study design.*** Eligible studies will include those that analysed or presented qualitative data by considering principles of human factors or ergonomics, systems engineering, sociotechnical or socioecological systems, or other relevant theories or frameworks, which enabled the identification of at least one of the following concepts: work system elements ( *e.g.*, people, environments, tasks, tools, technologies), work processes ( *e.g.*, obtaining supply, administering, monitoring), work outcomes ( *e.g.*, humanistic, organisational, clinical or economic) and factors (facilitators and barriers to safe and effective medicines management) associated with the work system.

### Information sources

Five databases will be used to retrieve relevant literature, namely MEDLINE, Embase, PsycInfo, CINAHL and Web of Science.

### Search strategy

The search strategy, shown in Table 1, was constructed for the MEDLINE and Embase databases using the Embase interface and was endorsed by a subject librarian. The systematic search will be tailored to the interface for PsycINFO, the Cumulative Index to Nursing and Allied Health Literature (CINAHL), and Web of Science.

**Table 1.** Combined search strategy adapted for the MEDLINE and Embase databases through the Embase interface.

Concepts (combined with the ‘AND’ operator)	Search terms
**Laypeople**	lay:ab,ti,kw OR layperson$:ab,ti,kw OR laypeople:ab,ti,kw OR ((lay NEAR/1 people$):ti,ab,kw) OR patient$:ti,ab,kw OR sufferer$:ti,ab,kw OR carer$:ab,ti,kw OR partner$:ti,ab,kw OR family:ti,ab,kw OR families:ti,ab,kw OR 'laypeople'/exp OR 'caregiver'/exp OR 'layperson'/exp OR 'patient'/exp OR ‘family’/exp OR ‘family relation’/exp
**Non-professional** ** settings**	household*:ab,ti,kw OR domicil*:ab,ti,kw OR dwelling$:ti,ab,kw OR apartment$:ti,ab,kw OR house$:ti,ab,kw OR ((personal NEAR/1 space$):ti,ab,kw) OR abode*:ti,ab,kw OR home$:ti,ab,kw OR residence$:ti,ab,kw OR ((non NEAR/1 professional$ NEAR/1 setting$):ti,ab,kw) OR ‘household’/exp OR ‘community dwelling person’/exp OR ‘personal space’/exp OR ‘residence’/exp
**Medication**	medication$:ab,ti,kw OR medicine$:ab,ti,kw OR drug$:ab,ti,kw OR pharmacotherap*:ab,ti,kw OR prescription$:ti,ab,kw OR (over NEAR/2 counter):ti,ab,kw OR remedy:ti,ab,kw OR remedies:ti,ab,kw OR 'drug therapy'/exp OR 'drug administration'/exp OR 'prescription'/exp OR 'prescription drug'/exp OR 'non prescription drug'/exp
**Study design: data to be** ** presented or analysed ** **using human factors and** ** ergonomics perspectives**	((system$ NEAR/1 engineering NEAR/1 initiative$ NEAR/1 for NEAR/1 patient$ NEAR/1 safety):ab,ti,kw) OR SEIPS:ti,ab,kw OR ((structure$ NEAR/1 process NEAR/1 outcome$):ti,ab,kw) OR ((system$ NEAR/1 thinking): ti,ab,kw) OR ((system$ NEAR/1 analys?s):ab,ti,kw) OR ((human NEAR/1 factor$):ab,ti,kw) OR ergonomic$: ab,ti,kw OR ((work NEAR/1 system$):ab,ti,kw) OR ((medication NEAR/1 work):ab,ti,kw) OR sociotechnical: ab,ti,kw OR ((socio NEAR/1 technical):ti,ab,kw) OR ((socio NEAR/1 ecological):ti,ab,kw) OR socioecological: ti,kw,ab OR macroergonomic$:ab,ti,kw OR mesoergonomic$:ab,ti,kw OR microergonomic$:ab,ti,kw OR ((system$ NEAR/1 engineering):ab,ti,kw) OR barrier$:ab,ti,kw OR impediment$:ab,ti,kw OR obstacle$: ti,ab,kw OR facilitator$:ti,ab,kw OR enabler$:ti,ab,kw OR 'human factor'/exp OR 'human factors'/exp OR 'human factors research'/exp OR 'human factors engineering'/exp OR 'ergonomics'/exp OR 'sociotechnical system'/exp OR ‘socioecological model’/exp OR ‘systems theory’/exp OR ‘system analysis’/exp OR 'macroergonomics'/exp OR 'barriers'/exp OR ‘obstacles’/ exp OR 'facilitator'/exp

Each search string will focus on four key concepts: 1) medication use; 2) by a population of patients or informal carers; 3) within non-formal settings; as reported by 4) studies related to the field of HFE which can provide details around work systems, processes and outcomes. The four strings will be combined with the ‘AND’ operator to yield relevant results.

### Selection of studies

Search results from each database will be uploaded to EndNote (Clarivate, London, UK) and de-duplicated. This body of citations will be imported to the Covidence systematic review software (Veritas Health Innovation, Melbourne, Australia), where further de-duplication and screening will be performed. All reviewers will independently review a proportion of titles and abstracts to ensure a common understanding of the application of eligibility criteria. In round one, two reviewers will then independently screen each study by title and abstract. Selected studies from round one will advance to round two where two reviewers will independently screen the article full text to determine final eligibility. Where differences in opinion arise or when a decision cannot be made at either round one or two, consensus will be reached ideally by discussion or through arbitration by another reviewer. A PRISMA flow diagram detailing study selection will be presented in the review ^26^.

### Data charting process

The review will employ the SEIPS model ^17^ to structure the charting of reported work system elements (persons, tasks, tools, internal and external environments, organisation), work processes and work outcomes.

A predefined list of elements, shown below, will be used to chart data ^25^. Data charting will be piloted on three included studies independently by two authors and will be iteratively adapted to ensure all relevant items are collected ^25^. One reviewer will then undertake data charting on the remaining studies. When 10% of studies are charted, a cross-check will be performed by a second reviewer to ensure all meaningful semantic units were charted and classified appropriately, and to enable early diagnosis and repair of issues. If inconsistencies arise, a subsequent cross-check will be performed when 25% of all studies are charted. Should discrepancies persist, all included studies will be cross-checked.

The data charting form will record the following elements, which will be analysed to meet the corresponding review objectives ^25^:

1. Study characteristics including country and date of publication, study aims, design, sample size and participant characteristics,
2. Methodological details related to the use of conceptual or theoretical frameworks or methods of data analysis or synthesis,
3. Data that have been analysed or presented from a human factors or systems-based perspective, and which relate to medication self-management as practised by patients and informal carers in non-formal settings. This includes data relevant to work systems, processes, outcomes and related factors (facilitators and barriers),
4. Reported knowledge gaps and recommendations for future work.

### Data analysis

Descriptive statistics will be used to characterise the body of studies ( *e.g.*, date and place of publication and participant demographics) and will be carried out using Microsoft Excel (Microsoft Corporation, Redmond, Washington). Further, the analytical approaches applied in the included studies will be described.

Basic deductive qualitative content analysis ^25^, directed by the SEIPS model, will be employed to report a list of the work system elements, interactions and outcomes reported in the body of studies, thereby exploring medication self-management as practiced by patients and informal carers. This will also facilitate the identification of research gaps in the current body of evidence and opportunities for future research. An appropriate data analysis/management tool *e.g.*, Microsoft Excel or NVivo (QSR International) will be used to support qualitative data analysis and management.

Included studies will be assessed during data charting in terms of their richness (adequacy of data in the included studies) and relevance (the degree of fit of the included studies to our review questions). Borrowing from the realist paradigm and guidance for assessing the confidence in evidence from reviews of qualitative research ^29^, a five-point richness scale will be used to estimate the depth of theoretical application of human factors theories and perspectives, and a five-point relevance scale will be used to determine how relevant each study is to our review objectives. This assessment of richness and relevance will complement the above-mentioned method in meeting the objective to identify research gaps and direct future studies.

This deductive coding approach may not accommodate all charted data, as some may fall outside of SEIPS categories, including data that can help contextualise phenomena ^30^. Nevertheless, this can be addressed by accommodating inductive approaches that are sensitive enough, especially with respect to subsequent analyses of these data.

### Data presentation

Charted data units will be aggregated and analysed. Findings related to the categorisation of data will be presented in a tabulated form for each research objective. Gaps in the available body of evidence and opportunities for future research will be identified by deductively coding the data using SEIPS and then mapping the aggregated coded data against SEIPS ^25^. Descriptive summaries will accompany each resulting data set and will respond to each research question.

### Risk of bias and quality assessment

Consistent with guidance for scoping reviews, which aim to describe, summarise and present available data ^25^, quality or risk of bias assessments about how well each included study addressed its objective will not be performed.

### Research ethics committee approval

Approval from a research ethics committee is not necessary in line with scoping review methodology, because the data being used are available in the public domain.

### Study status

The literature search has been completed and screening by title and abstract is underway.

## Discussion

This scoping review aims to describe and categorise the available evidence about non-formal medication self-management work systems. Using the SEIPS model ^17^, the scoping review will map the available evidence to describe each concept *e.g.*, work system elements, the relevant processes and resulting outcomes, the relevance and the richness of the available data to address the research objectives, and will facilitate the identification of research gaps and opportunities for future work. The findings will inform whether a future systematic review or primary studies would best contribute to the construction or validation of a framework to support research and development about a systems-based analysis of non-formal medication self-management.

The proposed review has a number of strengths. The suggested PPCI approach will enhance the likelihood that patients and their representatives are at the centre of the review, that the research is relevant and meaningful and that it will be utilised to inform subsequent research, policy or practice. Adherence to the ACTIVE framework ^22^ will enhance the PPCI approach employed. The review team have experience across the relevant fields of medication management, medication safety, human factors and ergonomics, and systematic reviewing. The convergence of these and the PPCI experiences will enhance the rigour of the data charting and interpretation.

## Data Availability

No data are associated with this article. Zenodo: PRISMA-P checklist for ‘The Medication Self-Management Work System of Patients and Informal Carers from a Human Factors & Ergonomics Perspective: A Scoping Review Protocol’. https://doi.org/10.5281/zenodo.7463641 ^28^. Data are available under the terms of the Creative Commons Attribution 4.0 International license (CC-BY 4.0).
