# Supplementary material for: The medication self-management work system of patients and informal carers from a human factors & ergonomics perspective: A scoping review protocol
Source: HRB Open Res. 2023 Jan 10;6:4. [Version 1] doi: 10.12688/hrbopenres.13674.1 (PMC10311124; doi:10.12688/hrbopenres.13674.1)
Supplement: Peer review history

# Peer review: Reviewer response for version 1

**DOI:** 10.21956/hrbopenres.14953.r34444

**Published:** 2023-08-16

**Authors:** Adam B Sutherland

**Affiliations:** 1 The University of Manchester, Manchester, England, UK

Thanks so much for allowing me to review this protocol for a scoping review relating to the potential harms associated with the increased reliance on patients and carers to medication-related work in non-clinical settings. 

 It's certainly an interesting field for study, and a scoping review using HFE methods is welcomed. I have just a couple of comments and reflections.

1. While you state that you will involve patients and carers in the project, you have not described HOW they would be identified or recruited.  I note that a previous reviewer has asked if they have been involved in the identification of the question therefore my comment stems from this. Who are your potential PPCI representatives, how were they identified and recruited, and how will you support them in this endeavour?
2. Your search strategy is specifically seeking papers with a HFE focus, and my worry is that you're limiting the review from the start.  HFE is still relatively new in healthcare, and newer still in healthcare outside of formal care environments.  There is a limited number of theoretically sound studies in this field in hospitals, and even fewer in non-clinical settings.  I would not be surprised if you identified a relatively small number of papers, all clustered from 2010 onwards.  I note that you state that the literature search has already been conducted.  However if you find a paucity of studies in your initial sweep, I would recommend broadening your search criteria to include qualitative observational studies of patient/carer medication work WITHOUT an HFE approach.
3. I struggle with the conceptual "othering" of patients and carers in the field of medication work.  As other reviewers have pointed out, there are elements of the medication process where patients and professionals co-exist, and my concerns come from our "work as imagined" and "work as prescribed" and the potential for undervaluing the importance of the patient's "work as done."  Patients and carers have agency and autonomy, and what we classify as "errors" are in many cases rational decisions taken to accommodate the medication into day to day life (see both the May and Maidment citations provided by Reviewer 2).  How will your protocol support the identification of and separation of these two concepts?  

Is the study design appropriate for the research question?

Partly

Is the rationale for, and objectives of, the study clearly described?

Yes

Are sufficient details of the methods provided to allow replication by others?

No

Are the datasets clearly presented in a useable and accessible format?

Not applicable

Reviewer Expertise:

Paediatric medicines safety; Human Factors and Ergonomics; Complex Systems and Theory Experience-Based Co-Production;

I confirm that I have read this submission and believe that I have an appropriate level of expertise to confirm that it is of an acceptable scientific standard, however I have significant reservations, as outlined above.

# Peer review: Reviewer response for version 1

**DOI:** 10.21956/hrbopenres.14953.r34443

**Published:** 2023-06-29

**Authors:** Tilo Mentler

**Affiliations:** 1 Trier University of Applied Sciences, Trier, Germany

This study protocol describes how to conduct a scoping review of lay medication use in settings that are not directly healthcare settings. I thank the authors for their contribution and careful drafting. Likewise, the systemic human factors approach is to be appreciated.

 The goal and necessity of the study are understandable, but individual justifications are perhaps a bit brief. It has long been common practice for patients to take medications (in part) at home. The arguments could certainly be worked out more clearly here.

 The authors state, " *Studies that have an element of professional or semi-professional service provision will be excluded.*" If, as is also stated, all phases of drug administration are considered, there should always be a professional medical part, at least in the case of prescription drugs. This should be clarified.

 The ACM Digital Library would also need to be considered, especially regarding technology-based approaches to medication management. There are also quite a few papers from the human factors area.

Is the study design appropriate for the research question?

Yes

Is the rationale for, and objectives of, the study clearly described?

Yes

Are sufficient details of the methods provided to allow replication by others?

Yes

Are the datasets clearly presented in a useable and accessible format?

Not applicable

Reviewer Expertise:

human computer interaction, safety-critical systems, human factors & ergonomics

I confirm that I have read this submission and believe that I have an appropriate level of expertise to confirm that it is of an acceptable scientific standard.

## References

[1] : The 'Self' as Barrier for Self-Management Technologies in Healthcare?. *PervasiveHealth '18: Proceedings of the 12th EAI International Conference on Pervasive Computing Technologies for Healthcare* .2018; 10.1145/3240925.3240974 336-346 10.1145/3240925.3240974 Reference source

# Peer review: Reviewer response for version 1

**DOI:** 10.21956/hrbopenres.14953.r33582

**Published:** 2023-04-12

**Authors:** Nina Fudge

**Affiliations:** 1 Wolfson Institute of Population Health, Queen Mary University of London, London, England, UK

Thank you for the opportunity to review this protocol paper of a scoping review on medication self-management work undertaken by patients and their informal carers. The authors’ proposal to frame the review using human factors and ergonomics approaches and systems thinking models provides a novel approach for exploring medication management in informal settings.

 The first sentence in the abstract and introduction is correct in that healthcare is increasingly being delivered closer to the patient’s homes (e.g. monitoring blood pressure at home, remote care delivery, etc.) but I think this link to medicine management and taking medicines at home needs a better explanation. Haven’t patients always taken their medication in the home? Could the authors please expand on how medicine management has become more burdensome because of the push to deliver care closer to home? Or has medicines management work become more burdensome because of the increase in prescribing behaviour and polypharmacy (as the authors allude to)?

 I understand that the focus is on medication management in the home/informal setting and the hazards this can create. But I was surprised not to see more mentioned about the source of medication which requires management - i.e. prescribing by health care professionals and the relationship between work as prescribed by professionals to patients and patients’ capacity to carry out this work (see the work of Carl May and colleagues ^1^, and Ian Maidment and colleagues ^2^). How will the authors address the treatment burden which is surely not solely the responsibility of patients?

 The review will adopt comprehensive patient, public and carer involvement, and from what I understood from the protocol, PPCI will occur from the study selection stage and onwards in the review process. But I wondered if patients and carers had been involved in the identification of the review questions and if so how their involvement shaped the resulting review.

Is the study design appropriate for the research question?

Yes

Is the rationale for, and objectives of, the study clearly described?

Yes

Are sufficient details of the methods provided to allow replication by others?

Yes

Are the datasets clearly presented in a useable and accessible format?

Yes

Reviewer Expertise:

Qualitative analysis, patient and public involvement, multiple long-term conditions, polypharmacy

I confirm that I have read this submission and believe that I have an appropriate level of expertise to confirm that it is of an acceptable scientific standard.

## References

[1] : Rethinking the patient: using Burden of Treatment Theory to understand the changing dynamics of illness. *BMC Health Services Research* .2014;14(1) : 10.1186/1472-6963-14-281 10.1186/1472-6963-14-281 24969758PMC4080515

[2] : Towards an understanding of the burdens of medication management affecting older people: the MEMORABLE realist synthesis. *BMC Geriatr* .2020;20(1) : 10.1186/s12877-020-01568-x 183 10.1186/s12877-020-01568-x 32498672PMC7272211
